# Supplementary material for: Resolving the full spectrum of human genome variation using Linked-Reads
Source: Genome Res. 2019 Apr;29(4):635–45. doi: 10.1101/gr.234443.118 (PMC6442396; doi:10.1101/gr.234443.118)
Supplement: Supplemental Material [file supp_gr.234443.118_Supplemental_longranger-2.2.zip › longranger-2.2.tar.bz2!/lr2.2-public/lib/stan/cmdstan/stan/doc/api/html/_eigen___num_traits_8hpp_source.html]

Stan: src/stan/agrad/rev/matrix/Eigen\_NumTraits.hpp Source File


|  |  |
| --- | --- |
|  | Stan  2.5.0  probability, sampling & optimization |


- Main Page
- Related Pages
- Namespaces
- Classes
- Files

- File List
- File Members

All Classes Namespaces Files Functions Variables Typedefs Enumerations Enumerator Friends Macros Pages

- src
- stan
- agrad
- rev
- matrix

Eigen\_NumTraits.hpp

Go to the documentation of this file.

1 #ifndef STAN\_\_AGRAD\_\_REV\_\_MATRIX\_\_EIGEN\_NUMTRAITS\_HPP

2 #define STAN\_\_AGRAD\_\_REV\_\_MATRIX\_\_EIGEN\_NUMTRAITS\_HPP

3

4 #include <limits>

5 #include <stan/math/matrix/Eigen.hpp>

6 #include <stan/agrad/rev/var.hpp>

7

8 namespace stan {

9  namespace agrad {

10

11  namespace {

12  class gevv\_vvv\_vari : public stan::agrad::vari {

13  protected:

14  stan::agrad::vari\* alpha\_;

15  stan::agrad::vari\*\* v1\_;

16  stan::agrad::vari\*\* v2\_;

17  double dotval\_;

18  size\_t length\_;

19  inline static double eval\_gevv(const stan::agrad::var\* alpha,

20  const stan::agrad::var\* v1, int stride1,

21  const stan::agrad::var\* v2, int stride2,

22  size\_t length, double \*dotprod) {

23  double result = 0;

24  for (size\_t i = 0; i < length; i++)

25  result += v1[i\*stride1].vi\_->val\_ \* v2[i\*stride2].vi\_->val\_;

26  \*dotprod = result;

27  return alpha->vi\_->val\_ \* result;

28  }

29  public:

30  gevv\_vvv\_vari(const stan::agrad::var\* alpha,

31  const stan::agrad::var\* v1, int stride1,

32  const stan::agrad::var\* v2, int stride2, size\_t length) :

33  vari(eval\_gevv(alpha,v1,stride1,v2,stride2,length,&dotval\_)), length\_(length) {

34  alpha\_ = alpha->vi\_;

35  v1\_ = (stan::agrad::vari\*\*)stan::agrad::memalloc\_.alloc(2\*length\_\*sizeof(stan::agrad::vari\*));

36  v2\_ = v1\_ + length\_;

37  for (size\_t i = 0; i < length\_; i++)

38  v1\_[i] = v1[i\*stride1].vi\_;

39  for (size\_t i = 0; i < length\_; i++)

40  v2\_[i] = v2[i\*stride2].vi\_;

41  }

42  virtual ~gevv\_vvv\_vari() {}

43  void chain() {

44  const double adj\_alpha = adj\_ \* alpha\_->val\_;

45  for (size\_t i = 0; i < length\_; i++) {

46  v1\_[i]->adj\_ += adj\_alpha \* v2\_[i]->val\_;

47  v2\_[i]->adj\_ += adj\_alpha \* v1\_[i]->val\_;

48  }

49  alpha\_->adj\_ += adj\_ \* dotval\_;

50  }

51  };

52  }

53

54  }

55 }

56

57

58 namespace Eigen {

59

64  template <>

65  struct NumTraits<stan::agrad::var> {

71  typedef stan::agrad::var Real;

72

78  typedef stan::agrad::var NonInteger;

79

85  typedef stan::agrad::var Nested;

86

93  inline static Real epsilon() {

94  return std::numeric\_limits<double>::epsilon();

95  }

96

100  inline static Real dummy\_precision() {

101  return 1e-12; // copied from NumTraits.h values for double

102  }

103

110  inline static Real highest() {

111  return std::numeric\_limits<double>::max();

112  }

113

120  inline static Real lowest() {

121  return -std::numeric\_limits<double>::max();

122  }

123

128  enum {

129  IsInteger = 0,

130  IsSigned = 1,

131  IsComplex = 0,

132  RequireInitialization = 0,

133  ReadCost = 1,

134  AddCost = 1,

135  MulCost = 1,

136  HasFloatingPoint = 1

137  };

138  };

139

140  namespace internal {

144  template<>

145  struct significant\_decimals\_default\_impl<stan::agrad::var,false>

146  {

147  static inline int run()

148  {

149  using std::ceil;

150  using std::log;

151  return cast<double,int>(ceil(-log(std::numeric\_limits<double>::epsilon())

152  / log(10.0)));

153  }

154  };

155

160  template <>

161  struct scalar\_product\_traits<stan::agrad::var,double> {

162  typedef stan::agrad::var ReturnType;

163  };

164

169  template <>

170  struct scalar\_product\_traits<double,stan::agrad::var> {

171  typedef stan::agrad::var ReturnType;

172  };

173

177  template<typename Index, bool ConjugateLhs, bool ConjugateRhs>

178  struct general\_matrix\_vector\_product<Index,stan::agrad::var,ColMajor,ConjugateLhs,stan::agrad::var,ConjugateRhs>

179  {

180  typedef stan::agrad::var LhsScalar;

181  typedef stan::agrad::var RhsScalar;

182  typedef typename scalar\_product\_traits<LhsScalar, RhsScalar>::ReturnType ResScalar;

183  enum { LhsStorageOrder = ColMajor };

184

185  EIGEN\_DONT\_INLINE static void run(

186  Index rows, Index cols,

187  const LhsScalar\* lhs, Index lhsStride,

188  const RhsScalar\* rhs, Index rhsIncr,

189  ResScalar\* res, Index resIncr, const ResScalar &alpha)

190  {

191  for (Index i = 0; i < rows; i++) {

192  res[i\*resIncr] += stan::agrad::var(new stan::agrad::gevv\_vvv\_vari(&alpha,((int)LhsStorageOrder == (int)ColMajor)?(&lhs[i]):(&lhs[i\*lhsStride]),((int)LhsStorageOrder == (int)ColMajor)?(lhsStride):(1),rhs,rhsIncr,cols));

193  }

194  }

195  };

196  template<typename Index, bool ConjugateLhs, bool ConjugateRhs>

197  struct general\_matrix\_vector\_product<Index,stan::agrad::var,RowMajor,ConjugateLhs,stan::agrad::var,ConjugateRhs>

198  {

199  typedef stan::agrad::var LhsScalar;

200  typedef stan::agrad::var RhsScalar;

201  typedef typename scalar\_product\_traits<LhsScalar, RhsScalar>::ReturnType ResScalar;

202  enum { LhsStorageOrder = RowMajor };

203

204  EIGEN\_DONT\_INLINE static void run(

205  Index rows, Index cols,

206  const LhsScalar\* lhs, Index lhsStride,

207  const RhsScalar\* rhs, Index rhsIncr,

208  ResScalar\* res, Index resIncr, const RhsScalar &alpha)

209  {

210  for (Index i = 0; i < rows; i++) {

211  res[i\*resIncr] += stan::agrad::var(new stan::agrad::gevv\_vvv\_vari(&alpha,((int)LhsStorageOrder == (int)ColMajor)?(&lhs[i]):(&lhs[i\*lhsStride]),((int)LhsStorageOrder == (int)ColMajor)?(lhsStride):(1),rhs,rhsIncr,cols));

212  }

213  }

214  };

215  template<typename Index, int LhsStorageOrder, bool ConjugateLhs, int RhsStorageOrder, bool ConjugateRhs>

216  struct general\_matrix\_matrix\_product<Index,stan::agrad::var,LhsStorageOrder,ConjugateLhs,stan::agrad::var,RhsStorageOrder,ConjugateRhs,ColMajor>

217  {

218  typedef stan::agrad::var LhsScalar;

219  typedef stan::agrad::var RhsScalar;

220  typedef typename scalar\_product\_traits<LhsScalar, RhsScalar>::ReturnType ResScalar;

221  static void run(Index rows, Index cols, Index depth,

222  const LhsScalar\* \_lhs, Index lhsStride,

223  const RhsScalar\* \_rhs, Index rhsStride,

224  ResScalar\* res, Index resStride,

225  const ResScalar &alpha,

226  level3\_blocking<LhsScalar,RhsScalar>& /\* blocking \*/,

227  GemmParallelInfo<Index>\* /\* info = 0 \*/)

228  {

229  for (Index i = 0; i < cols; i++) {

230  general\_matrix\_vector\_product<Index,LhsScalar,LhsStorageOrder,ConjugateLhs,RhsScalar,ConjugateRhs>::run(

231  rows,depth,\_lhs,lhsStride,

232  &\_rhs[((int)RhsStorageOrder == (int)ColMajor)?(i\*rhsStride):(i)],((int)RhsStorageOrder == (int)ColMajor)?(1):(rhsStride),

233  &res[i\*resStride],1,alpha);

234  }

235  }

236  };

237  }

238 }

239

240 #endif

Eigen::internal::general\_matrix\_vector\_product< Index, stan::agrad::var, ColMajor, ConjugateLhs, stan::agrad::var, ConjugateRhs >::RhsScalar

stan::agrad::var RhsScalar

**Definition:** Eigen\_NumTraits.hpp:181

Eigen::internal::general\_matrix\_vector\_product< Index, stan::agrad::var, RowMajor, ConjugateLhs, stan::agrad::var, ConjugateRhs >::ResScalar

scalar\_product\_traits< LhsScalar, RhsScalar >::ReturnType ResScalar

**Definition:** Eigen\_NumTraits.hpp:201

Eigen::internal::general\_matrix\_vector\_product< Index, stan::agrad::var, ColMajor, ConjugateLhs, stan::agrad::var, ConjugateRhs >::LhsScalar

stan::agrad::var LhsScalar

**Definition:** Eigen\_NumTraits.hpp:180

v1\_

stan::agrad::vari \*\* v1\_

**Definition:** Eigen\_NumTraits.hpp:15

Eigen::internal::general\_matrix\_matrix\_product< Index, stan::agrad::var, LhsStorageOrder, ConjugateLhs, stan::agrad::var, RhsStorageOrder, ConjugateRhs, ColMajor >::LhsScalar

stan::agrad::var LhsScalar

**Definition:** Eigen\_NumTraits.hpp:218

Eigen::NumTraits< stan::agrad::var >::highest

static Real highest()

Return standard library's highest for double-precision floating point, std::numeric\_limits<double>max...

**Definition:** Eigen\_NumTraits.hpp:110

Eigen::internal::scalar\_product\_traits< stan::agrad::var, double >::ReturnType

stan::agrad::var ReturnType

**Definition:** Eigen\_NumTraits.hpp:162

Eigen::NumTraits< stan::agrad::var >::Real

stan::agrad::var Real

Real-valued variables.

**Definition:** Eigen\_NumTraits.hpp:71

Eigen::internal::general\_matrix\_matrix\_product< Index, stan::agrad::var, LhsStorageOrder, ConjugateLhs, stan::agrad::var, RhsStorageOrder, ConjugateRhs, ColMajor >::run

static void run(Index rows, Index cols, Index depth, const LhsScalar \*\_lhs, Index lhsStride, const RhsScalar \*\_rhs, Index rhsStride, ResScalar \*res, Index resStride, const ResScalar &alpha, level3\_blocking< LhsScalar, RhsScalar > &, GemmParallelInfo< Index > \*)

**Definition:** Eigen\_NumTraits.hpp:221

Eigen::internal::scalar\_product\_traits< double, stan::agrad::var >::ReturnType

stan::agrad::var ReturnType

**Definition:** Eigen\_NumTraits.hpp:171

stan::math::max

double max(const double a, const double b)

**Definition:** max.hpp:7

stan::length

size\_t length(const T &)

**Definition:** traits.hpp:159

stan::agrad::vari::val\_

const double val\_

The value of this variable.

**Definition:** vari.hpp:37

stan::agrad::memalloc\_

memory::stack\_alloc memalloc\_

**Definition:** var\_stack.cpp:16

Eigen::internal::general\_matrix\_vector\_product< Index, stan::agrad::var, RowMajor, ConjugateLhs, stan::agrad::var, ConjugateRhs >::LhsScalar

stan::agrad::var LhsScalar

**Definition:** Eigen\_NumTraits.hpp:199

Eigen::internal::significant\_decimals\_default\_impl< stan::agrad::var, false >::run

static int run()

**Definition:** Eigen\_NumTraits.hpp:147

Eigen::internal::general\_matrix\_vector\_product< Index, stan::agrad::var, RowMajor, ConjugateLhs, stan::agrad::var, ConjugateRhs >::run

static EIGEN\_DONT\_INLINE void run(Index rows, Index cols, const LhsScalar \*lhs, Index lhsStride, const RhsScalar \*rhs, Index rhsIncr, ResScalar \*res, Index resIncr, const RhsScalar &alpha)

**Definition:** Eigen\_NumTraits.hpp:204

stan::agrad::ceil

fvar< T > ceil(const fvar< T > &x)

**Definition:** ceil.hpp:15

Eigen::internal::general\_matrix\_vector\_product< Index, stan::agrad::var, ColMajor, ConjugateLhs, stan::agrad::var, ConjugateRhs >::ResScalar

scalar\_product\_traits< LhsScalar, RhsScalar >::ReturnType ResScalar

**Definition:** Eigen\_NumTraits.hpp:182

Eigen::NumTraits< stan::agrad::var >::lowest

static Real lowest()

Return standard library's lowest for double-precision floating point, &#45;std::numeric\_limits<double...

**Definition:** Eigen\_NumTraits.hpp:120

stan::agrad::var::vi\_

vari \* vi\_

Pointer to the implementation of this variable.

**Definition:** var.hpp:40

Eigen::NumTraits< stan::agrad::var >::Nested

stan::agrad::var Nested

Nested variables.

**Definition:** Eigen\_NumTraits.hpp:85

stan::math::rows

size\_t rows(const Eigen::Matrix< T, R, C > &m)

**Definition:** rows.hpp:12

v2\_

stan::agrad::vari \*\* v2\_

**Definition:** Eigen\_NumTraits.hpp:16

stan::agrad::vari

The variable implementation base class.

**Definition:** vari.hpp:28

Eigen::NumTraits< stan::agrad::var >::NonInteger

stan::agrad::var NonInteger

Non-integer valued variables.

**Definition:** Eigen\_NumTraits.hpp:78

Eigen::internal::general\_matrix\_matrix\_product< Index, stan::agrad::var, LhsStorageOrder, ConjugateLhs, stan::agrad::var, RhsStorageOrder, ConjugateRhs, ColMajor >::ResScalar

scalar\_product\_traits< LhsScalar, RhsScalar >::ReturnType ResScalar

**Definition:** Eigen\_NumTraits.hpp:220

Eigen::internal::general\_matrix\_vector\_product< Index, stan::agrad::var, RowMajor, ConjugateLhs, stan::agrad::var, ConjugateRhs >::RhsScalar

stan::agrad::var RhsScalar

**Definition:** Eigen\_NumTraits.hpp:200

var.hpp

stan::math::cols

size\_t cols(const Eigen::Matrix< T, R, C > &m)

**Definition:** cols.hpp:12

Eigen.hpp

stan::agrad::var

Independent (input) and dependent (output) variables for gradients.

**Definition:** var.hpp:27

stan::math::e

double e()

Return the base of the natural logarithm.

**Definition:** constants.hpp:86

Eigen::internal::general\_matrix\_matrix\_product< Index, stan::agrad::var, LhsStorageOrder, ConjugateLhs, stan::agrad::var, RhsStorageOrder, ConjugateRhs, ColMajor >::RhsScalar

stan::agrad::var RhsScalar

**Definition:** Eigen\_NumTraits.hpp:219

Eigen::internal::general\_matrix\_vector\_product< Index, stan::agrad::var, ColMajor, ConjugateLhs, stan::agrad::var, ConjugateRhs >::run

static EIGEN\_DONT\_INLINE void run(Index rows, Index cols, const LhsScalar \*lhs, Index lhsStride, const RhsScalar \*rhs, Index rhsIncr, ResScalar \*res, Index resIncr, const ResScalar &alpha)

**Definition:** Eigen\_NumTraits.hpp:185

Eigen::NumTraits< stan::agrad::var >::dummy\_precision

static Real dummy\_precision()

Return dummy precision.

**Definition:** Eigen\_NumTraits.hpp:100

Eigen::NumTraits< stan::agrad::var >::epsilon

static Real epsilon()

Return standard library's epsilon for double-precision floating point, std::numeric\_limits<double>::e...

**Definition:** Eigen\_NumTraits.hpp:93

stan::agrad::log

fvar< T > log(const fvar< T > &x)

**Definition:** log.hpp:15

dotval\_

double dotval\_

**Definition:** Eigen\_NumTraits.hpp:17

length\_

size\_t length\_

**Definition:** Eigen\_NumTraits.hpp:18

stan::agrad::vari::adj\_

double adj\_

The adjoint of this variable, which is the partial derivative of this variable with respect to the ro...

**Definition:** vari.hpp:43

alpha\_

stan::agrad::vari \* alpha\_

**Definition:** Eigen\_NumTraits.hpp:14


---

[ Stan Home Page ]

*© 2011–2014,
Stan Development Team.*
